# Emerging Issues From a Global Overview of Digital Covid-19 Certificate Initiatives

**DOI:** 10.3389/fpubh.2021.744356

**Published:** 2021-11-19

**Authors:** Fidelia Cascini, Francesco Andrea Causio, Giovanna Failla, Andriy Melnyk, Valeria Puleo, Luca Regazzi, Walter Ricciardi

**Affiliations:** ^1^Section of Hygiene and Public Health, Department of Life Sciences and Public Health, Università Cattolica del Sacro Cuore, Roma, Italy; ^2^Department of Public Health, University of Verona, Verona, Italy; ^3^Scientific Direction, Istituti Clinici Scientifici Maugeri, Pavia, Italy

**Keywords:** digital certificate, pandemic (COVID-19), interoperability, standards, gateway (GW)

## Introduction

International mobility is one of the highest priorities of the current globalized society after being greatly hampered by the Covid-19 pandemic. Worldwide, much effort has been put into revitalizing non-essential travel (e.g., for tourism), while also preserving safety. In fact, an increasing number of national and international initiatives have been launched to facilitate a mutual recognition of Covid-19 status certificates amongst countries^1^.

Countries have been invited by the World Health Organization (WHO) to adopt an omni-comprehensive risk-based approach. This is based on lifting public health measures, such as testing and/or quarantine requirements, and requires travelers to be fully vaccinated with WHO-approved vaccines, have documented proof of past infection, or to have been tested negative to WHO-approved rRT-PCR tests or antigen detection rapid diagnostic tests (Ag-RDTs)^2^.

However, the rapid increase of Covid-19 certificates worldwide is showing differences in the governance of issuing and validation processes and has raised challenges such as the reliability of the certificates and trustworthiness of certifying bodies or authorities, the data protection guarantees, and the interoperability prospects.

Since February 2021, the WHO has been promoting the use of the International Certificate of Vaccination and Prophylaxis (ICVP) to record the SARS-CoV-2 vaccination proof. It proposed a solution to leverage public key infrastructures (i.e., the same used for credit card transactions) to establish a cryptographically supported trust framework for Covid-19 certificates. This would be used between member states, with the WHO in the role of trust broker^3^.

However, different Digital Covid-19 Certificates (DCC) are currently available worldwide, possibly affecting international mobility for safe people.

## Digital Covid-19 Certificate Initiatives

On May 31, 2021, the European Commission proposed to update the Council Recommendation on the coordination of free movement limitations in the European Union (EU). Clear rules were established on the conditions for lifting travel restrictions for the people who hold the European DCC. Two months later, on July 1, this DCC came into effect to facilitate safe travel within the EU, with the possibility to include Iceland, Liechtenstein, Norway, Switzerland, San Marino, and Vatican City^4^. The European DCC is currently the most advanced and complete Covid-19 certificate worldwide; it is the only multilaterally certificate that has been adopted and recognized by several countries within a harmonized framework. It includes three different types of certificates: the vaccination certificate, the SARS-CoV-2 negative test certificate, and the Covid-19 recovery certificate. The European DCC allows people to avoid tests on arrival or mandatory quarantine when people move from one country to another. It contains personal key information such as name and date of birth, issuing member state, and unique identifier code (QR code) of the certificate.

Regarding the vaccination certificate, it is limited to vaccines authorized by the European Medicines Agency (EMA) and reports the vaccine product, manufacturer, number of doses performed, and the date(s) of vaccine inoculation. For the SARS-CoV-2 negative test certificate, the type of test, date and time, the center which performed the test, and the result are included. Accepted tests are NAAT tests (including RT-PCR tests) and rapid antigen tests, named in the list established by the Council Recommendation 2021/C 24/01. Self-tests are not included as they are not performed under controlled conditions^5^. Digital Covid-19 Certificates holders can enter European countries without restrictions, even if their certificate contains proof of recovery from Covid-19 infection. For this certificate, the date of positive test results, the certificate issuer, the date of issue, and the date of validity are reported.

The DCC format is the same for all European countries, but the rules imposed by each member state to allow the entry are different. For instance, it is up to the single member state the choice of admitting vaccination certificates concerning vaccines not authorized by the EMA. A central EU gateway, an information technology service through which the DCC signatures are controlled across the member states, has been created but certificate information cannot be retained by the countries of destination. Personal data is stored in the member state that issued the DCC. Each member state has been supported by the EU during the national software and app development phases for issuing, archiving, and verifying certificates as well as during the test phase, which is necessary for gateway integration.

Other, non-EU countries are working on their own DCC, and are at different stages of maturity. China^6^ was the first country to roll out a Covid-19 international health digital certificate in March 2021. Named the “International Travel Health Certificate,” it allows internal travel between cities and regions and boarding on flights to China. However, both travelers who are Chinese citizens and who are non-citizens flying to China are required to take an IgM antibody test and a molecular test within 48 h of boarding their flights in addition to having a valid vaccination certificate. Available both on paper and on the WeChat mini-app “Health Code App (international version),” it contains the result of the molecular test, serum IgG antibody test, vaccination status, and personal data all encrypted by a QR code. The same app is also used as a contact tracing app with a traffic light-like green, yellow, or red QR code based on the assessment of a person's individual risk of being infected by SARS-CoV-2, based on people met and places visited. The green code is officially sanctioned as valid for traveling, but different cities and provinces issue their own codes, thus requiring a traveler to apply for a green code when moving between different regions within China. While Chinese citizens must submit the test results through the WeChat app to obtain the green health code, foreigners must also obtain a QR code, named the “Health Declaration Code,” by uploading their test results to the designated website.

In early April 2021, the United States White House Press Secretary issued a statement regarding the possibility of a DCC, declaring that there will be no attempts at a federal US Digital Vaccination Pass, as it would require a federal vaccination database. According to both Republicans and the Biden administration, after nationwide waves of protests, a Vaccination Pass would force Americans to carry a health-based ID, which might lead to instances of discrimination and unfair treatment. Consequently, the federal government left the initiative up to private, non-profit organizations, and individual states. The paper cards issued by the Center for Disease Control and Prevention (CDC) in regard to vaccination status offer little in terms of unique identification (such as a QR Code) and in terms of data trust, making them easy to forge. Thus far, the state of New York is the only one offering a DCC, called excelsior pass, where a system of paired phone apps—one for scanning/verifying and the other for showing the pass—allows individuals to show their vaccination status or negative test results. Organizers of venues, large gatherings, and sporting events can thus enforce Covid-19 status requirements for safety^7^.

In Australia^8^^,^^9^, people who have received both the doses of the approved vaccines (Vaxzevira by AstraZeneca or Comirnaty by Pfizer-BioNTech), can access the digital proof of their full vaccination. This is automatically^9^ generated and available on the Express Plus Medicare App or the myGov website (even for those who opted out of My Health Record, since the Australian Immunization Register is a separate database). Those experiencing issues can require an immunization history statement from a vaccine provider or the Australian Immunization Register. Currently, this certificate doesn't allow holders to avoid travel or domestic restrictions and only serves as evidence of vaccination status.

Israel^10^, which has been looked at for a long time as the country with the best vaccine rollout model, issued a Ministry of Health-approved DCC. Anyone who was vaccinated or recovered from Covid-19 was eligible for receiving it. For people aged 16 or younger or those having contraindications to the Covid-19 vaccine (i.e., unvaccinated and not planning to be vaccinated), the DCC was available for 72 h after having tested negative for SARS-CoV-2. This certificate was considered a solution to last until the end of 2020, but on June 1^st^, 2021, Israel retired its DCC system and now allows access to activities such as attendance at sports events, restaurants, and more to both vaccinated and unvaccinated citizens alike.

With the exception of the US, all of the above-mentioned countries decided to adopt centralized certification systems for Covid-19 immunity, but that is not the only possible solution. The Republic of San Marino introduced a decentralized verification system, in part based on blockchain technology. It is an immutable network of *blocks* of data whose copies are distributed across multiple entities or locations, thus making it a truly decentralized operation storage system. It allows operational transparency whilst maintaining individual privacy (as sensitive data blocks can be encrypted and accessed only by specific entities with an encryption key) and is paired with its centralized national vaccination database. Specifically, San Marino introduced a double certification system to ensure total interoperability with the technological standards used by the European Union while also adding a universal certification method based on non-fungible tokens, unique certificates of digital authenticity registered on the VeChainThor public blockchain^11^. This certificate is fully aligned with European standards: it is issued to certify a partial/completed vaccination course or a negative molecular/antigenic test or a negative serologic test or a previous infection from SARS-CoV-2 and contains a unique identifier associated with a QR code, as well as information about the name, surname, and date of birth of the person. The simplicity of blockchain-based verification allows San Marino's system to be highly portable outside of the EU, as the digital infrastructure on which it is based is already widespread.

## Discussion

**Figure 1:**
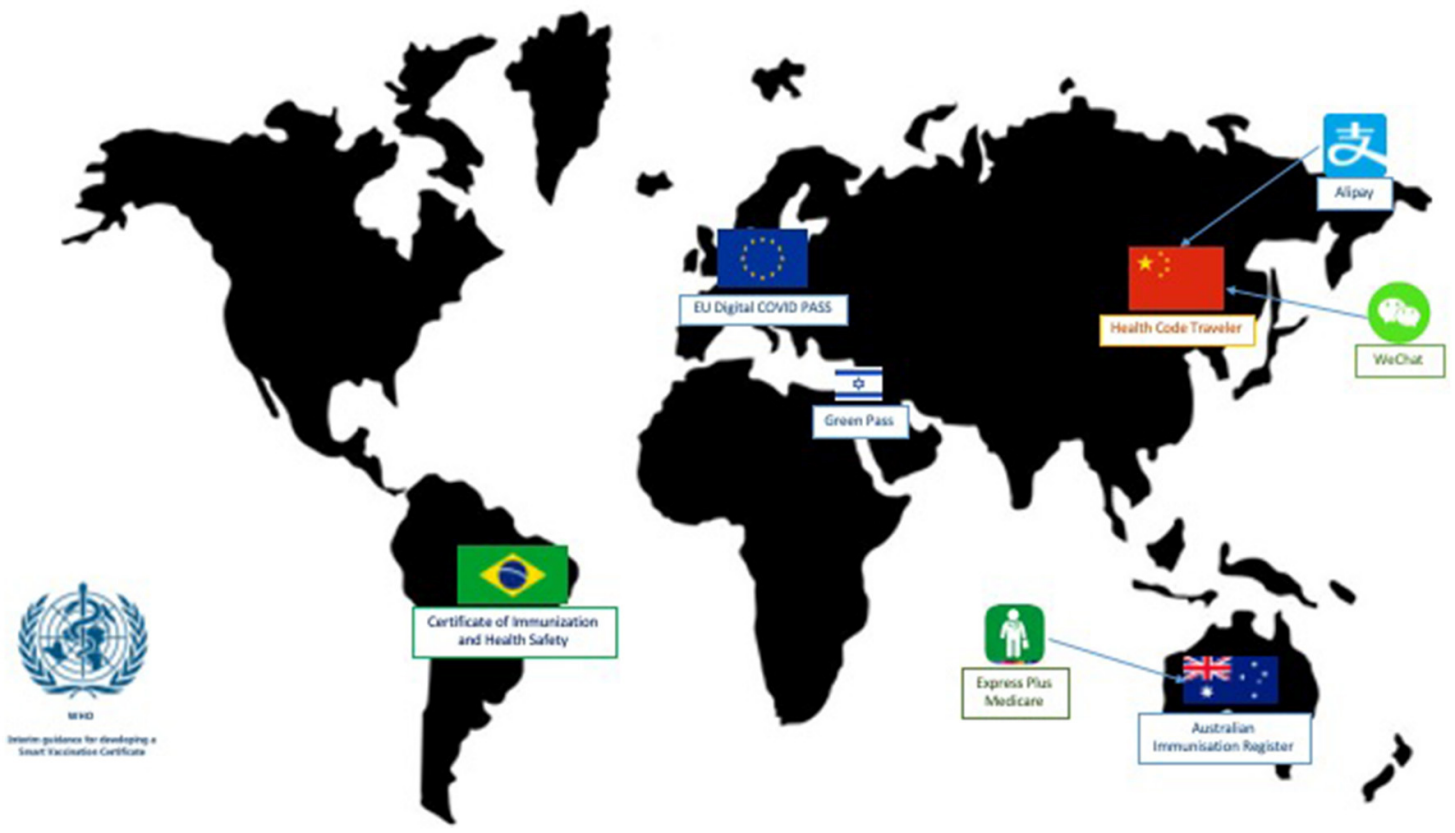
Digital Covid-19 certificates initiatives currently available worldwide.

Interesting DCC initiatives are currently available worldwide (Figure 1). However, despite the effort invested to facilitate international mobility for people, these technological solutions could be applied at a regional/local level, instead of solely addressing the challenge of safer global mobility. The challenge of mobility at a regional/local level challenge is that it remains bound to traditional and more reliable restrictive measures such as quarantine and repetition of diagnostic tests (swabs) instead of vaccines or digital recovery certifications. The main issues of this near-missed opportunity are two-fold: (1) the governance and universal acceptability of the issuing and verification process of the certificates; (2) the information included in the DCC and related privacy and ethical issues.

In terms of the first point, the reliability of vaccine, recovery, and test certificates is greatly variable among countries around the globe. It depends on authorized vaccines and diagnostic tests, and on bodies or professionals issuing recovery, vaccination, or test certificates. Despite the effort to create a homogeneous and egalitarian European DCC, different approaches still emerge from EU member states. Even when the type of authorized vaccines and/or the list of admitted rapid tests are the same for all the EU countries, the timing of vaccination and the validity of the starting and expiration time of test and recovery certificates differ from EU country to EU country. As examples, France currently requires 4 weeks from the second dose of vaccines to enter in the country while Austria and Czech Republic require 3 weeks from the first dose with 3 months of validity. In terms of the duration of the recovery certificate, most of the EU member states recognize 6 months of validity while Greece and Italy allow 9 months. Concerning the negative diagnostic test results, in general, rapid antigenic test are admitted (48 h) by EU countries while the Netherlands only accepts molecular tests. Regarding the governance of the DCC issuing and verification processes, most EU countries have the Ministry of Health as the public authority responsible for these activities. Further, the process to ensure a trust architecture to make possible that a DCC from Non-EU countries are verified in a common way, is still ongoing. Business rules for validation purposes should be the logical conditions used to assess the conformance of each certificate to the requirements of each country. However, the lack of harmonisation of verification rules is leading to different results for the same certificate, depending on the European member states' app verifying non-EU country DCCs. Digital Covid-19 Certificates from Non-EU countries accepted by the DCC European gateway are not automatically paired with DCC from EU member states accepted by non-EU countries involved.

This issue can get even more complex. China, for instance, accepts its national vaccines for its International Travel Health Certificate and recently started accepting some Western-made vaccines, such as Johnson and Johnson, Moderna, and Pfizer^12^. On the other hand, the EU is still evaluating whether to accept vaccines made in China, which have been used by many developing countries in their vaccination campaigns. These vaccines are currently under rolling review by the EMA, which does not recognize AstraZeneca vaccines made in India. Regarding this, the EU regulation allows individual EU countries to apply their own rules for vaccines not approved by the EMA: some countries such as Belgium, Germany, and Switzerland authorize people to enter if they received non-EU-endorsed vaccines, whereas others, such as Italy and France, don't allow that^13^.

Regarding the issue around information included in the DCC, privacy, and security issues remain core to the current discussion on the DCC. A universally accepted DCC has not been developed, for instance, in the United States of America since the requirements to show the proof of vaccination could violate privacy laws. On the other hand, the paper cards that can be used to verify an individual's vaccination status, as approved by the Center for Disease Control and Prevention (CDC), does not present a unique identifier or marker, such as the QR code used in Europe, making them prime targets for forgery^14^. Further, ethical problems are possibly exacerbating inequality in those countries where a universal health coverage is not conceived as part of the healthcare system. An immunity passport implicitly puts economic, social, and civil limitations on those who cannot obtain it (i.e., poor population groups that don't have the possibility to access vaccination and testing). A perverse incentive that may arise in those excluded from their country's workforce could be to actively seek infection in order to procure a Covid-19 certificate, potentially exposing others to health risks^15^.

Following huge internal pressures, Israel lifted vaccination-related restrictions, eliminating DCC privileges and allowing access to public places and events to vaccinated and unvaccinated people alike^16^. Israel's decision followed a long national debate where the DCC initiative was accused as being coercive and ambiguous legislation allowed employers and business owners to decide whether to require workers or clients to get a DCC or not^17^.

The way toward a safe and free global mobility during the pandemic remains far away, despite advanced digital solutions being available and suitable to overcome interoperability problems, as demonstrated by the Republic of San Marino. It is a good example of an integration opportunity where a decentralized verification system, based on blockchain technology and paired with a centralized national vaccination database, works by ensuring total interoperability with the technological standards used by the EU gateway and consistent with WHO standards.

## Author Contributions

FC and WR designed the paper. GF, VP, and AM searched the literature. FAC and LR selected information for data extraction. FC wrote the manuscript with the contribution of all the authors. All authors contributed to the article and approved the submitted version.

## Conflict of Interest

The authors declare that the research was conducted in the absence of any commercial or financial relationships that could be construed as a potential conflict of interest.

## Publisher's Note

All claims expressed in this article are solely those of the authors and do not necessarily represent those of their affiliated organizations, or those of the publisher, the editors and the reviewers. Any product that may be evaluated in this article, or claim that may be made by its manufacturer, is not guaranteed or endorsed by the publisher.

## Footnotes

^1^*Policy Considerations for Implementing a Risk-Based Approach to International Travel in the Context of Covid-19 - 02/07/2021*. Available online at: https://apps.who.int/iris/bitstream/handle/10665/342235/WHO-2019-nCoV-Policy-Brief-Risk-based-international-travel-2021.1-eng.pdf?sequence=1&isAllowed=y

^2^*Interim Guidance for Developing a Smart Vaccination Certificate - 19/03/2021*. Available online at: https://cdn.who.int/media/docs/default-source/documents/interim-guidance-svc_20210319_final.pdf?sfvrsn=b95db77d_11&download=true; *Interim Position Paper: Considerations Regarding Proof of Covid-19 Vaccination for International Travellers - 05/02/2021*. Available online at: https://www.who.int/news-room/articles-detail/interim-position-paper-considerations-regarding-proof-of-covid-19-vaccination-for-international-travellers; Available online at: https://www.ncbi.nlm.nih.gov/pmc/articles/PMC7198144/; *Policy Considerations for Implementing a Risk-Based Approach to International Travel in the Context of Covid-19 - 02/07/2021*. Available online at: https://apps.who.int/iris/bitstream/handle/10665/342235/WHO-2019-nCoV-Policy-Brief-Risk-based-international-travel-2021.1-eng.pdf?sequence=1&isAllowed=y

^3^Available online at: https://www.who.int/news-room/articles-detail/interim-position-paper-considerations-regarding-proof-of-covid-19-vaccination-for-international-travellers

^4^Available online at: https://ec.europa.eu/commission/presscorner/detail/en/qanda_21_1187

^5^Available online at: https://ec.europa.eu/info/live-work-travel-eu/coronavirus-response/safe-covid-19-vaccines-europeans/eu-digital-covid-certificate_en

^6^Available online at: https://www.loc.gov/item/global-legal-monitor/2021-03-18/china-digital-vaccination-certificate-for-citizens-traveling-overseas-launched/

^7^Available online at: https://covid19vaccine.health.ny.gov/excelsior-pass

^8^Available online at: https://www.covidpasscertificate.com/australians-support-vaccine-passport-travel/

^9^Available online at: https://www1.racgp.org.au/newsgp/clinical/how-does-australia-s-new-digital-vaccination-certificate

^10^Available online at: https://corona.health.gov.il/en/directives/green-pass-info/.

^11^Available online at: https://www.dnv.com/news/san-marino-approves-national-green-pass-allowing-citizens-and-residents-to-move-freely-203736; https://www.consigliograndeegenerale.sm/on-line/home/streaming-video-consiglio/documento17125439.html

^12^Available online at: https://www.washingtonpost.com/world/asia_pacific/china-vaccine-passport-visas-covid/2021/04/20/8a6422d0-a14e-11eb-8a6d-f1b55f463112_story.html

^13^Available online at: https://apnews.com/article/europe-business-lifestyle-health-travel-f678a53270470d9b35ce6259e48548bf

^14^*Getting Your Covid-19 Vaccine*. Available online at: https://www.cdc.gov/coronavirus/2019-ncov/vaccines/expect.html

^15^*Covid-19 Immunity Passports and Vaccination Certificates: Scientific, Equitable, and Legal Challenges*. Available online at:https://www.sciencedirect.com/science/article/pii/S0140673620310345?via%3Dihub

^16^Available online at: https://www.nytimes.com/2021/02/18/world/middleeast/israel-covid-vaccine-reopen.html?searchResultPosition=4

^17^Available online at: https://www.nytimes.com/2021/04/05/world/middleeast/israel-vaccinations.html

